# The Rumen Microbiota Contributes to the Development of Mastitis in Dairy Cows

**DOI:** 10.1128/spectrum.02512-21

**Published:** 2022-02-16

**Authors:** Xiaoyu Hu, Shuang Li, Ruiying Mu, Jian Guo, Caijun Zhao, Yongguo Cao, Naisheng Zhang, Yunhe Fu

**Affiliations:** a Department of Clinical Veterinary Medicine, College of Veterinary Medicine, Jilin University, Changchun, Jilin Province, People's Republic of China; b Linqu County Animal Husbandry Development Center, Linqu, People's Republic of China; State Key Laboratory of Microbial Resources, Institute of Microbiology, Chinese Academy of Sciences

**Keywords:** SARA, rumen microbiota, mastitis, LPS, *Stenotrophomonas*

## Abstract

Mastitis, a highly prevalent disease in dairy cows, is commonly caused by local infection of the mammary gland. Our previous studies have suggested that the gut microbiota plays an important role in the development of mastitis in mice. However, the effects of rumen microbiota on bovine mastitis and the related mechanisms remain unclear. In this study, we assessed the effects and mechanisms of rumen microbiota on bovine mastitis based on the subacute rumen acidosis (SARA) model induced by feeding Holstein Frisian cows a high-concentrate diet for 8 weeks. Then, the inflammatory responses in the mammary gland and the bacterial communities of rumen fluid, feces, and milk were analyzed. The results showed that SARA induced mastitis symptoms in the mammary gland; activated a systemic inflammatory response; and increased the permeability of the blood-milk barrier, gut barrier, and rumen barrier. Further research showed that lipopolysaccharides (LPS), derived from the gut of SARA cows, translocated into the blood and accumulated in the mammary glands. Furthermore, the abundance of *Stenotrophomonas* was increased in the rumen of SARA cows, and mastitis was induced by oral administration of *Stenotrophomonas* in lactating mice. In conclusion, our findings suggested that mastitis is induced by exogenous pathogenic microorganisms as well as by endogenous pathogenic factors. Specifically, the elevated abundance of *Stenotrophomonas* in the rumen and LPS translocation from the rumen to the mammary gland were important endogenous factors that induced mastitis. Our study provides a foundation for novel therapeutic strategies that target the rumen microbiota in cow mastitis.

**IMPORTANCE** Mastitis is a common and frequently occurring disease of humans and animals, especially in dairy farming, which has caused huge economic losses and brought harmful substance residues, drug-resistant bacteria, and other public health risks. The traditional viewpoint indicates that mastitis is mainly caused by exogenous pathogenic bacteria infecting the mammary gland. Our study found that the occurrence of mastitis was induced by the endogenous pathway. Evidence has shown that rumen-derived LPS enters the mammary gland through blood circulation, damaging the blood-milk barrier and then inducing inflammation of the mammary gland in cows. In addition, a higher abundance of *Stenotrophomonas* in the rumen was closely associated with the development of mastitis. This study provides a basis for novel therapeutic strategies that exploit the rumen microbiota against mastitis in cows.

## INTRODUCTION

Milk production represents an important sector of the agricultural industry. Ruminant species are the primary animals that produce milk agriculturally, in which cows account for approximately 84% of total milk production worldwide (1). Mastitis, a major inflammatory symptom of the mammary gland, is one of the most serious diseases of dairy cows, and its occurrence has a massive negative effect on animal well-being and farm economics due to treatment costs and a reduction in milk production. It is commonly believed that the major cause of mastitis is invasion and colonization of the mammary gland by pathogens, such as Staphylococcus aureus, Streptococcus uberis, and Escherichia coli (2). However, recent studies have shown that 27.3% of cows that suffer from mastitis have culture-negative milk (3). Evidence has indicated that approximately 25% of milk samples from clinical mastitis are culture-negative or show no significant pathogens, and 30% of subclinical mastitis samples were reported to be-culture negative (3, 4). It is therefore possible that factors other than mastitis-associated pathogens are associated with the development of mastitis, such as rumen microbiota.

The rumen, as one of the most important digestive organs in ruminants, plays an important role in diseases of dairy cows. To maximize milk yield, cows are often fed a high-concentrate diet (HCD) to meet the nutritional demands of lactation. However, the long-term overfeeding of HCD often leads to a metabolic disorder termed subacute ruminal acidosis (SARA), which is mainly characterized by a prolonged decline in ruminal pH, causing rumen microbial imbalance (5). Epidemiological investigations have indicated a prevalence of SARA ranging from 18–40% in early- and mid-lactation dairy cows due to the addition of a high proportion of concentrate to the diet (6, 7), and most cases of mastitis take place in the early lactation phase (8). It has been suggested that the occurrence of mastitis in cows is associated with the development of SARA (9). In addition, SARA is a typical case model of rumen microflora disorder in cows, and changes in the rumen microbiota community may be associated with the development of cow mastitis (10). Recent studies have shown that the occurrence of mastitis is accompanied by changes in gut microbiota in dairy cows (11). Our previous studies also indicated that gut microbiota disturbance contributes to the development of mastitis induced by S. aureus or E. coli in mice (12, 13). Ma et al. (14) showed that fecal microbiota transplantation from cows with mastitis to germfree mice results in mastitis. This evidence indicated that the rumen microbiota is associated with the development of mastitis in dairy cows.

In addition, somatic cell count (SCC), consisting mostly of neutrophils, is the standard for the diagnosis of mastitis (15, 16). Neutrophil translocation from blood to milk must occur through the blood-milk barrier, which is an important gatekeeper that hinders the movement of molecules between the blood and organs (17). Evidence has shown that the gut microbiota plays an important role in maintaining the integrity of the blood–brain barrier (18), blood–testis barrier (19), and gut barrier (20). Our previous study also showed that gut microbiota disturbance increases inflammatory cell infiltration in the mammary gland by increasing the permeability of the blood–milk barrier in mice.

Thus, we speculated that the occurrence of mastitis is due both to infection by exogenous pathogenic microorganisms as well as endogenous factors; that is, the disturbance of the rumen bacterial community is an important inducer of mastitis in dairy cows. Therefore, we studied the correlations and mechanisms between rumen microbiota and mastitis of lactating dairy cows using a SARA model induced by HCD in the present study.

## RESULTS

### Establishment of the SARA model in dairy cows.

**FIG 1:**
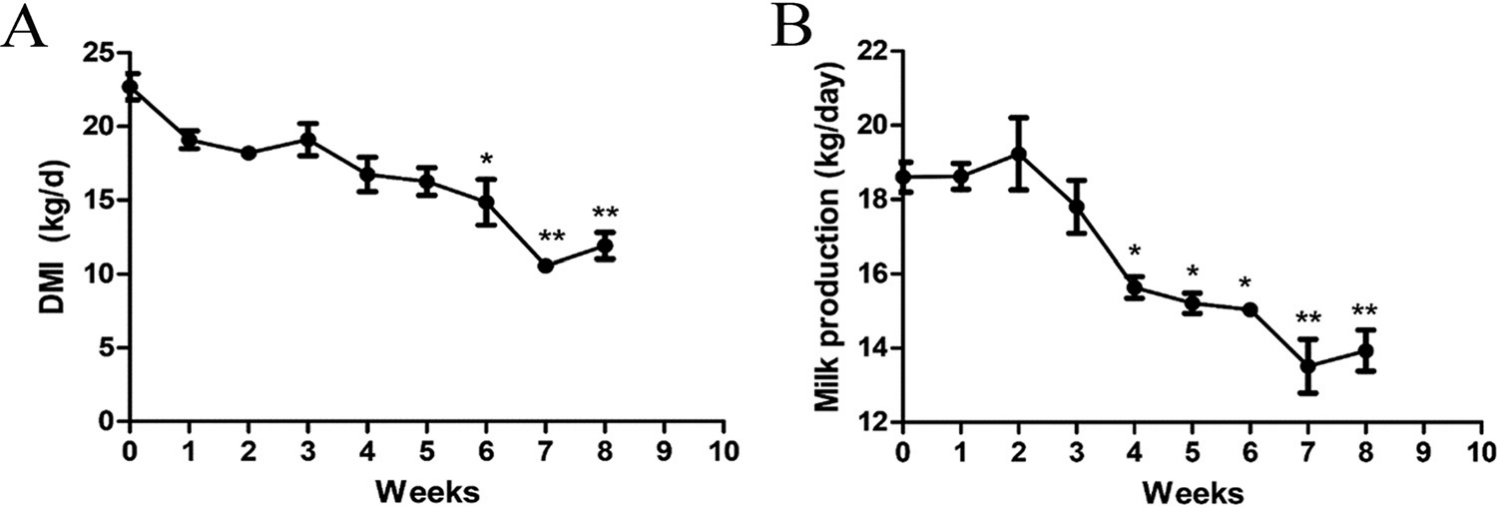
Feeding high-concentrate diet decreased the milk production and DMI. (A) DMI, and (B) milk production between control and SARA cows. *P *< 0.05 indicates a significant difference between the different groups.

To exclude the effect of experimental period on milk composition, we detected the milk composition of eight other Holstein Frisian cows with the same standards used to establish the SARA model (cows with similar lactation durability and body weight). The results showed that there were no significant changes in milk yield, milk fat, milk protein, lactose, urea nitrogen, or SCC of healthy cows during the trial period (see Supplementary Figure S1). To evaluate the HCD-induced SARA model in cows, the dry matter intake (DMI) and milk yield were measured weekly. After 8 weeks of feeding with HCD, the DMI and milk production were significantly reduced compared to the control groups (Fig. 1A and B). In addition, the pH of the rumen fluid was noticeably reduced, and a pH < 5.8 was sustained for more than 3 h at different periods of time in the cows fed an HCD for 8 weeks (Table S1 in the supplemental material), indicating that SARA model was efficiently established (21). In addition, the levels of pH in feces were also significantly lowered. However, there were no changes in the blood pH of SARA cows compared to the control cows (Table S1).

### SARA induces the inflammatory response in the mammary gland of dairy cows.

**FIG 2:**
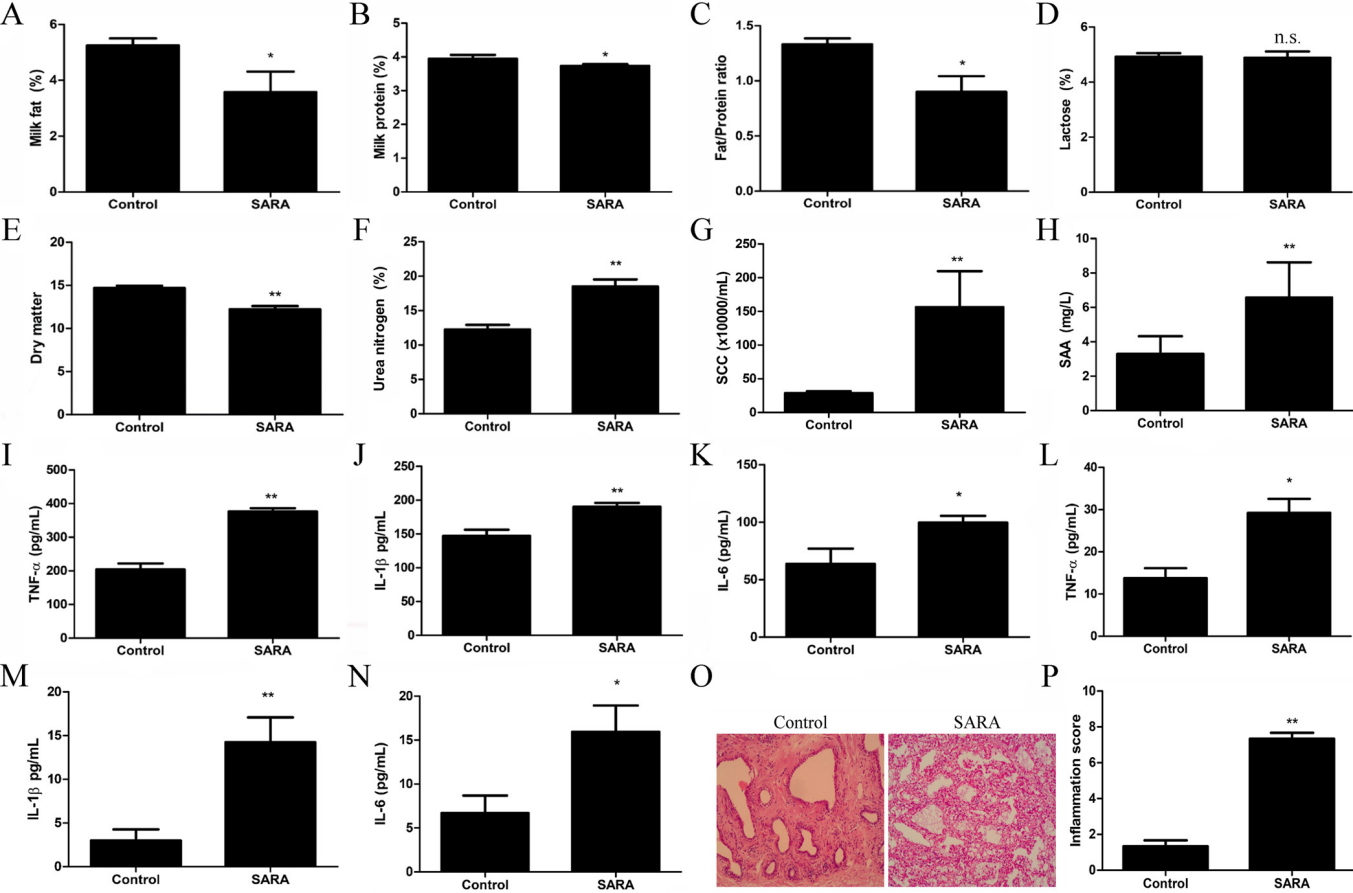
SARA induces the inflammatory response in the mammary gland. Zero and 8 weeks after feeding with HCD, the milk were collected for detecting of milk composition and inflammatory cytokines. (A) Milk fat, (B) milk protein, (C) fat/protein ratio, (D) lactose, (E) dry matter, (F) urea nitrogen, (G) somatic cell count (SCC), (H) SAA, (I) TNF-α, (J) IL-1β, (K) IL-6 levels in milk of control and SARA cows. (L) TNF-α, (M) IL-1β, (N) IL-6 in mammary gland of cows and SARA cows. (O) The hispathlogical changes, and (P) inflammation score in mammary gland of cows and SARA cows. *P *< 0.05 indicates a significant difference between the different groups.

Milk composition analysis showed that the contents of milk fat, milk protein, fat/protein ratio and dry matter were markedly decreased in SARA cows compared to the control cows (Fig. 2A, C–E). The content of lactose in milk from SARA cows was unchanged (Fig. 2D), and urea nitrogen was significantly increased compared to the milk from control cows (Fig. 2F). Importantly, SCC and serum amyloid A (SAA), two important indicators of mastitis, also significantly increased after cows suffered from SARA (Fig. 2G and H). In addition, the levels of proinflammatory cytokines, including tumor necros factor-α (TNF)-α, interleukin-1β (IL-1β), and interleukin-6 (IL-6), were clearly increased in the mammary gland (Fig. 2I–K) and in milk (Fig. 2L–N) from cows suffering from SARA. Furthermore, histopathological analysis of SARA cows showed a large amount of inflammatory cell infiltration, thickening of the alveolar wall, mammary gland destruction in the mammary gland and a higher inflammation score (Fig. 2O and P).

### SARA increases blood–milk barrier permeability in dairy cows.

**FIG 3:**
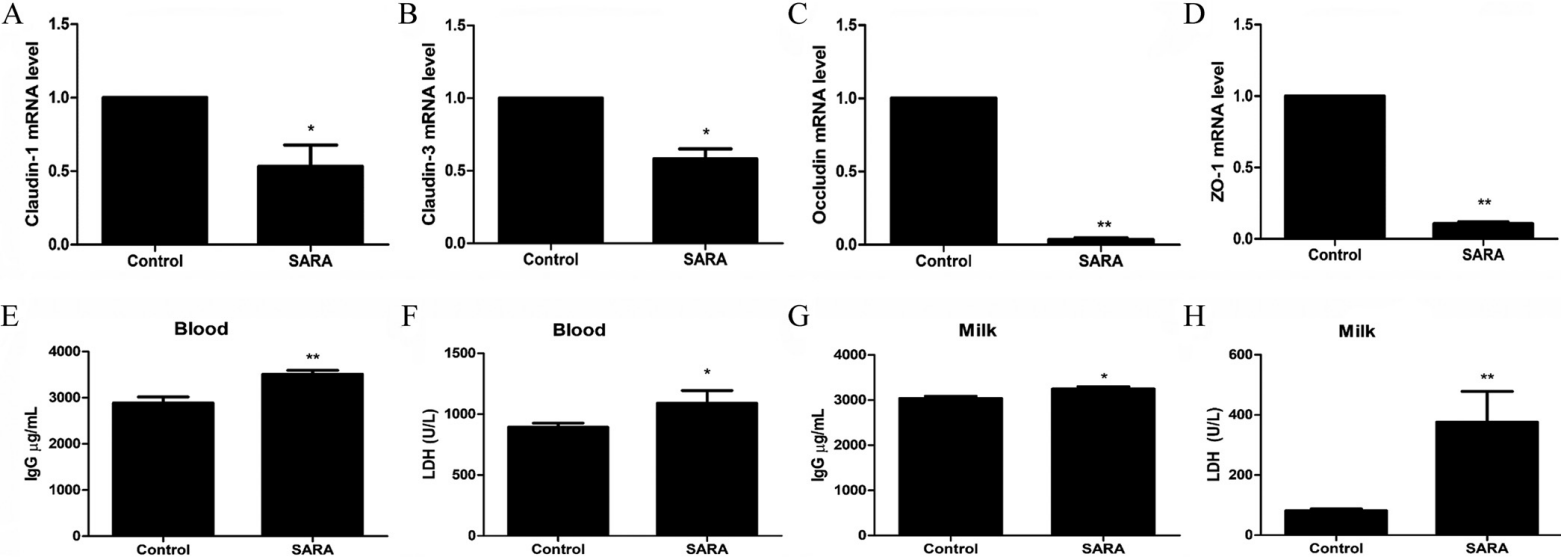
SARA increases blood-milk barrier permeability. Zero and 8 weeks after feeding with HCD, the mammary gland tissues were collected for detecting the expression of tight junction proteins Claudin-1, Claudin-3, Occludin, and ZO-1. In addition, the milk and blood were collected for detecting of LDH and IgG. The expression of tight junction proteins (A) Claudin-1, (B) Claudin-3, (C) Occludin, and (D) ZO-1 of rumen between control and SARA cows. (E) The concentration of IgG in plasma. (F) The concentration of LDH in plasma. (G) The concentration of IgG in milk. (H) The concentration of LDH in milk. *P *< 0.05 indicates a significant difference between the different groups.

Neutrophil translocation from blood to milk must occur through the blood-milk barrier, which is a specific structure that prevents foreign matter from entering the mammary gland from the blood or external environment (22). To evaluate the permeability of the blood-milk barrier, we detected the expression of tight junction proteins that make up the blood-milk barrier. The results showed that the expression of Claudin-1, Claudin-3, Occludin, and Zonula occludens-1 (ZO-1) was significantly reduced in SARA cows (Fig. 3A–D). In addition, blood-derived proteins in milk, including IgG and lactate dehydrogenase (LDH), serve as indicators of blood-milk barrier integrity (23). Moreover, the production of IgG and LDH was clearly increased in both the blood and milk of SARA cows compared to the control cows (Fig. 3E–H). These results suggested that SARA impairs the structure and function of the blood-milk barrier in dairy cows.

### Rumen-derived LPS may be associated with the development of mastitis in dairy cows.

**FIG 4:**
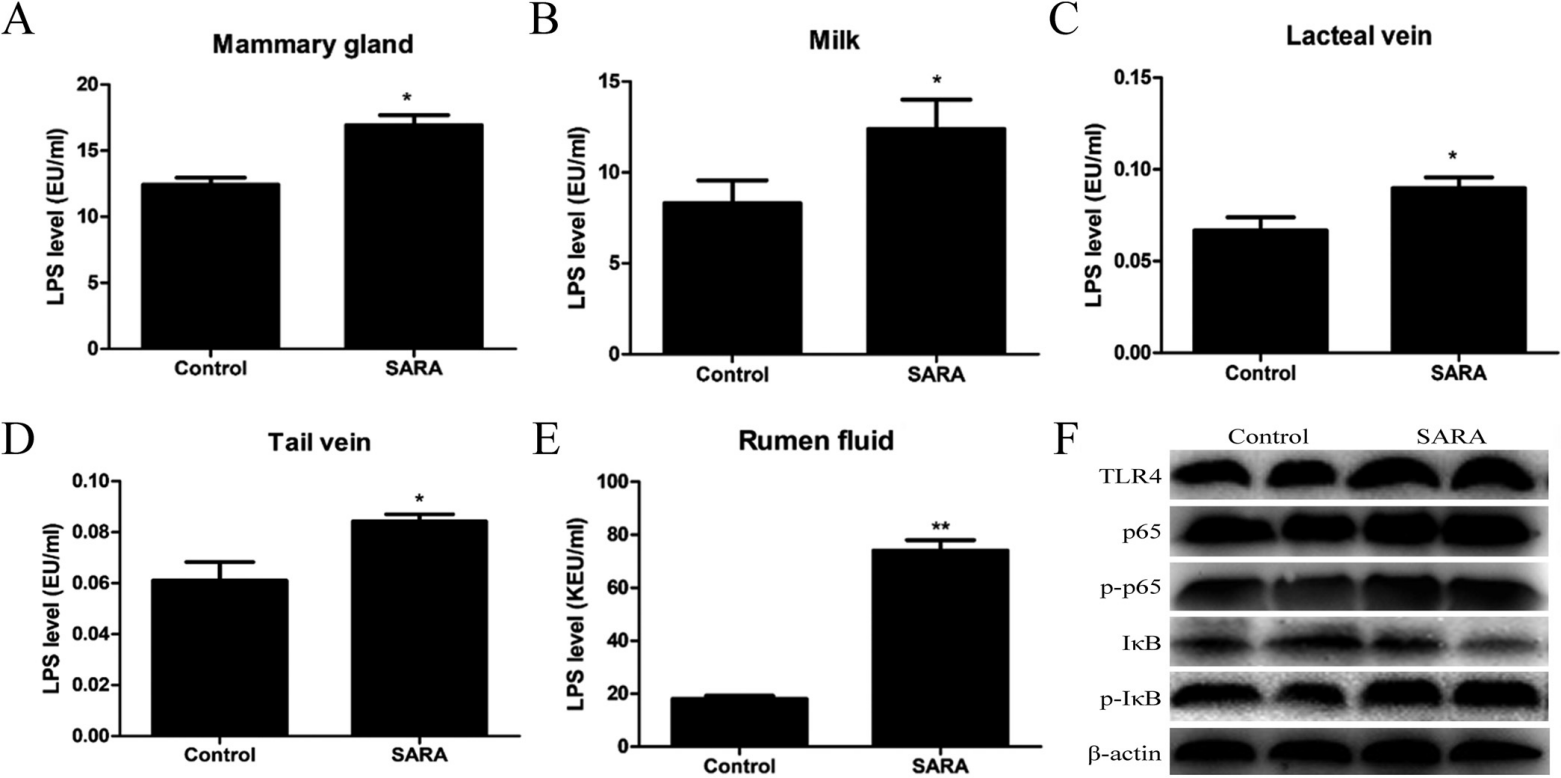
The levels of LPS and the expression of TLR4/NF-κB signaling pathway between control and SARA cows. Zero and 8 weeks after feeding with HCD, the mammary gland tissues, milk, lacteal vein, tail vein, and rumen fluid were collected and the levels of LPS were detected. In addition, the activation of TLR4/NF-κB signaling pathway were detected by Western blot. The concentration of (A) LPS from the mammary gland tissues, (B) milk, (C) lacteal vein, (D) tail vein, and (E) rumen fluid of control and SARA cows. (F) The activation of TLR4/NF-κB signaling pathway in the mammary gland of control and SARA cows. *P *< 0.05 indicates a significant difference between the different groups.

LPS, the main component of the cell walls of Gram-negative bacteria, is an important inflammatory substance leading to mastitis. Evidence has increasingly shown that LPS -derived from the rumen of SARA cows can translocate to the bloodstream. During the lactation period, the mammary gland blood volume of cows accounts for 8% of the total body blood volume. Therefore, we assessed whether cow mastitis induced by SARA was associated with LPS migration from the rumen to the mammary gland via the bloodstream. We found that the levels of LPS in the mammary gland, milk, lacteal veins, tail veins, and rumen fluid were all significantly increased in SARA cows (Fig. 4A–E). LPS is a strong activator of innate immune responses and is recognized by toll like receptor 4 (TLR4), which then activates nuclear factor-κB (NF-κB) to induce the transcription of TNF-α, IL-1β, and IL-6. The expression of TLR4, phosphorylated NF-κB p65, and phosphorylated inhibitor of NFκB was upregulated in the mammary glands of SARA cows compared with control cows (Fig. 4F). These results suggest that SARA of cows leads to rumen-derived LPS entering the mammary gland through blood circulation, damaging the blood–milk barrier, and inducing inflammation of the mammary gland in cows.

### SARA increases the permeability of the rumen and intestinal barrier.

**FIG 5:**
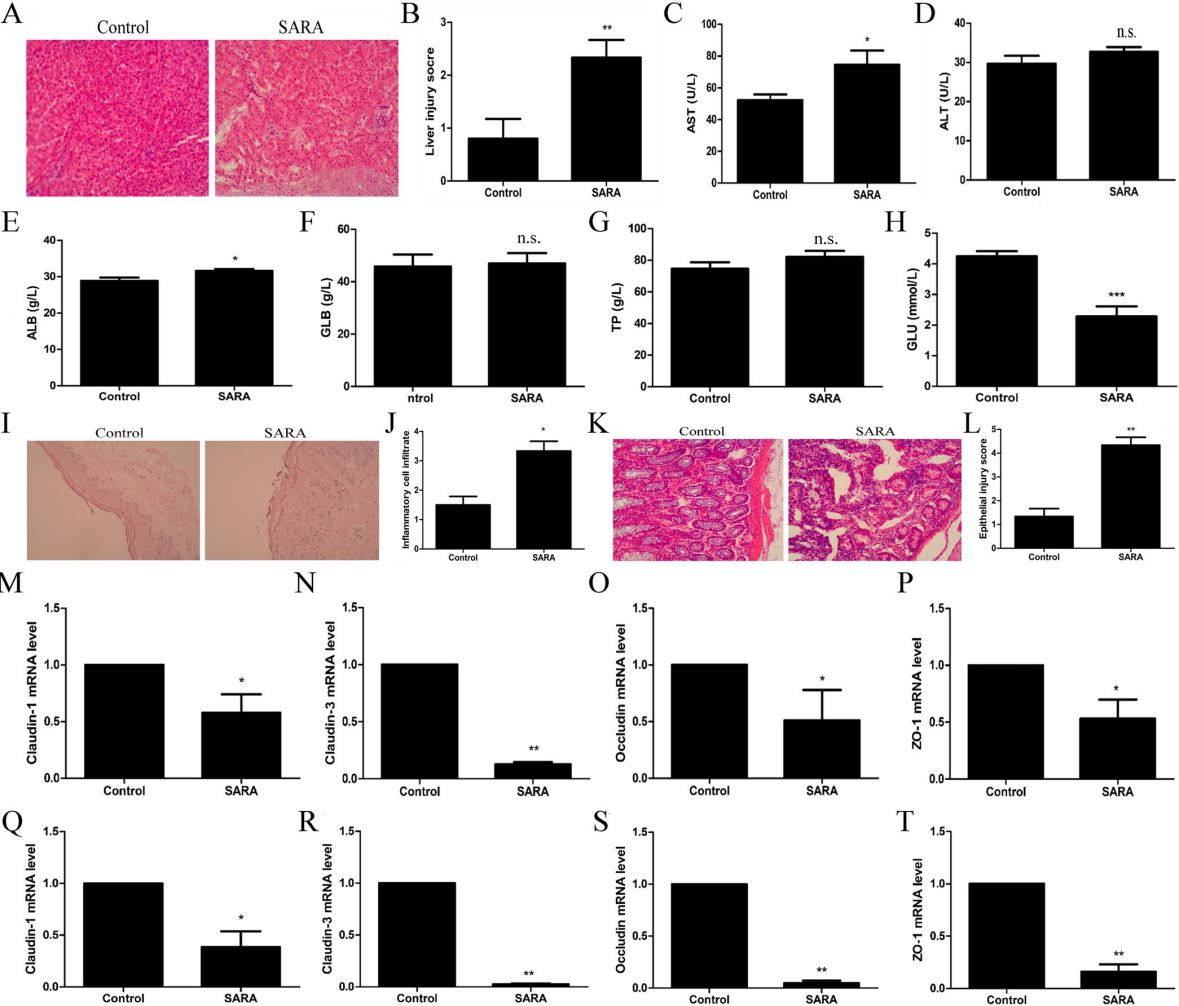
SARA increases rumen and gut barrier permeability. Zero and 8 weeks after feeding with HCD, the liver, rumen, and intestines were collected and the expression of tight junction protein were detected. In addition, the plasma was collected for detecting the concentration of AST, ALT, ALB, GLB, TP, and GLU. (A) The liver hispathological changes, and (B) liver injury score of control and SARA cows. (C) AST, (D) ALT, (E) ALB, (F) GLB, (G) TP, and (H) GLU concentration in plasma of control and SARA cows. (I) The rumen hispathological changes, and (J) inflammation score of the rumen between control and SARA cows. (K) The intestine hispathological changes, and (L) epithelial injury score of intestines between control and SARA cows. The expression of (M) Claudin-1, (N) Claudin-3, (O) Occludin, and (P) ZO-1 of rumen between control and SARA cows. The expression of (Q) Claudin-1, (R) Claudin-3, (S) Occludin, and (T) ZO-1 of intestines between control and SARA cows. *P*< 0.05 indicates a significant difference between the different groups.

The liver is an important metabolic and immune organ of ruminants, playing a vital role in transferring LPS to the bloodstream (24). In contrast, overproduction of LPS can also promote the occurrence of inflammatory responses and the aggregation of immune cells, thus interfering with the metabolism of substances in the liver (25). SARA cows showed severe pathological damage, including inflammatory cell infiltration and liver cell injury ballooning (Fig. 5A and B), and upregulated levels of AST (Fig. 5C). Plasma levels of ALB, GLOB, TP, and GLU were also assessed as indicators of the metabolism and internal organ status of animals (26). The data showed higher levels of ALB and lower levels of GLU, but no changes of GLOB and TP in plasma were observed in SARA cows (Fig. 5E–H). In addition, LPS entering the blood from the rumen needs to cross the rumen wall and/or intestinal tract; thus, rumen and gut epithelial permeability was assessed in cows from the control and SARA groups. The histological changes suggested that the rumen epithelium of SARA cows was incomplete, and greater numbers of immune cells infiltrated into the rumen epithelium of SARA cows (Fig. 5I and J). Furthermore, the detection of tight junction proteins of the rumen barrier indicated that the protein expression levels of Claudin-1, Claudin-3, Occludin, and ZO-1 were all significantly reduced in SARA cows compared to control cows (Fig. 5M–P). Moreover, histopathologic changes in intestinal tissues indicated gut epithelium desquamation and severe cellular damage in SARA cows compared to control cows, and SARA cows showed significantly higher epithelial damage scores in the intestine (Fig. 5K and L). In addition, the expression of Claudin-1, Claudin-3, Occludin, and ZO-1 in the intestinal barrier was significantly reduced in SARA cows compared to control cows (Fig. 5Q to T). These results suggested that SARA cows have increased permeability of the rumen and intestinal barrier, inducing the release of rumen-derived LPS into the bloodstream, and subsequent liver damage.

### Changes in the bacterial community in rumen fluid, feces, and milk induced by SARA.

**FIG 6:**
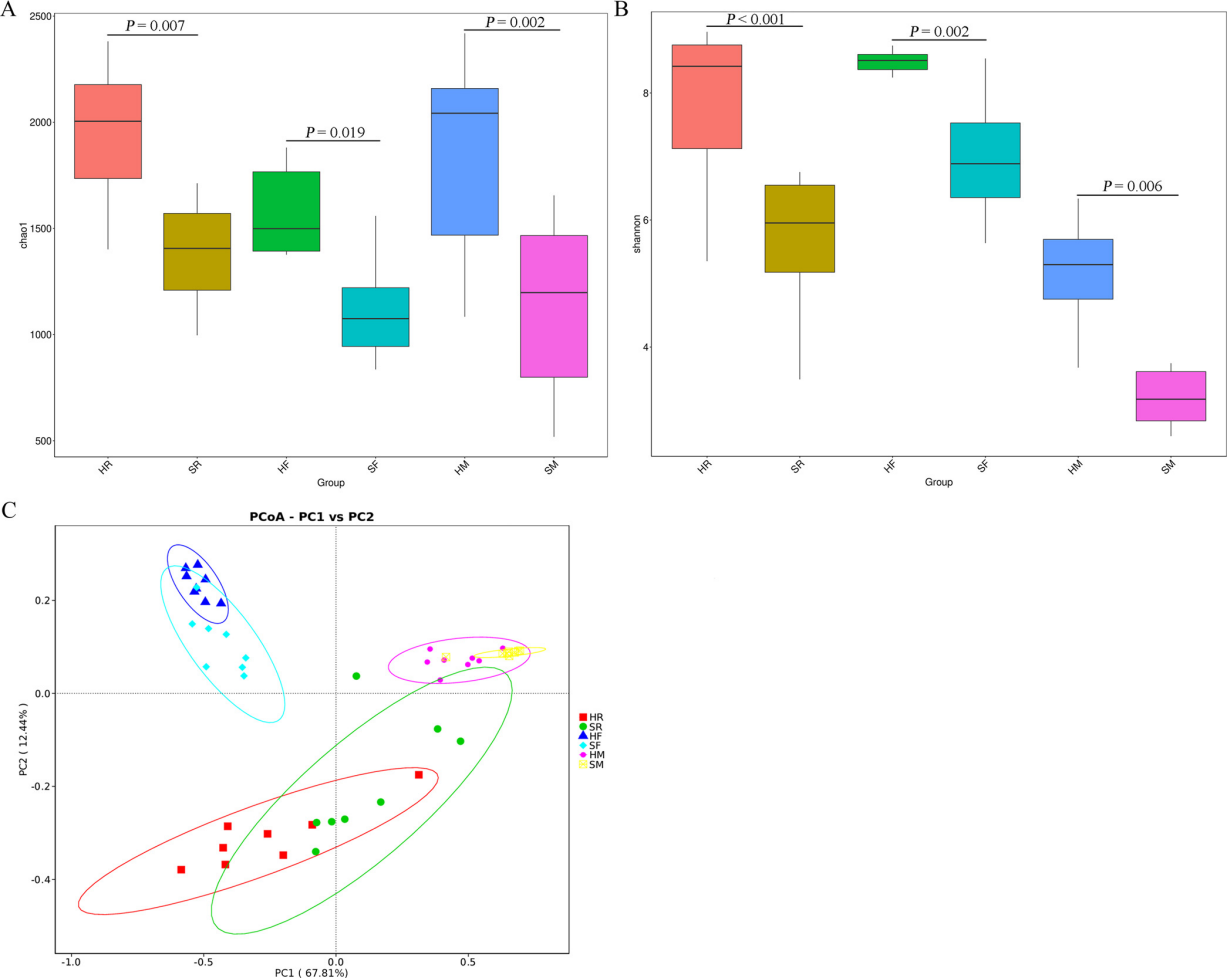
SARA diminished the richness and diversity of the rumen fluid, milk, and fecal microbiota. Zero and 8 weeks after feeding with HCD, the rumen fluid, milk, and feces samples were collected for analysis bacterial community. Comparison of the microbiota richness in terms of (A) chao 1 in milk, rumen fluid, and feces between control and SARA cows. Comparison of the microbiota diversity in terms of the (B) shannon index in milk, rumen fluid, and feces between control and SARA cows. (C) PCoA based on the weighted-UniFrac distance matrices in milk microbiota, rumen fluid microbiota, and feces microbiota between control and SARA cows. *P *< 0.05 indicates a significant difference between the different groups.

Milk, rumen fluid, and fecal samples from the same 8 Holstein Frisian cows on day 0 and week 8 after feeding an HCD were used to detect the microbiota community using PCR amplification of the V4 region of the bacterial 16S rRNA gene from all 48 samples. The 16S rRNA sequencing resulted in 3,456,642 Taxon Tags, with an average of 72,013 Taxon Tags per sample. For operational taxonomic units (OTUs) at a 3% distance, 9,495 different phylotypes were detected among all of the samples. Rarefaction curves for 48 samples indicated that the sampling depth was sufficient to characterize the majority of bacterial diversity (see Supplementary Figure S2 in the supplemental material). To explore whether mastitis induced by SARA was associated with changes in the microbiota, the bacterial richness and diversity in rumen fluid, feces, and milk were compared between control and SARA cows. The results showed that the estimators of community richness (Chao 1 and ACE) and diversity (Shannon index and Simpson) of milk, rumen fluid, and feces in SARA cows were significantly reduced compared to the control cows (Fig. 6A and B; see Supplementary Figure S3). In addition, a principal coordinate analysis (PCoA) plot, based on the weighted- UniFrac distance matrices, indicated that the microbiota composition of rumen fluid, feces, and milk from control cows was clearly separated from that of SARA cows (Fig. 6C).

**FIG 7:**
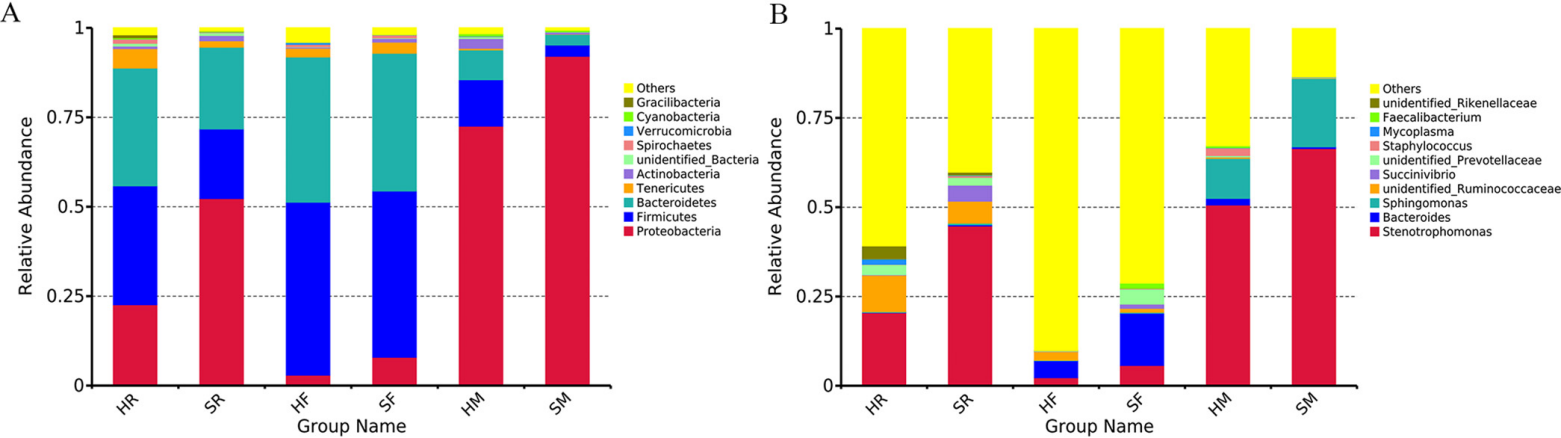
Comparisons at the phylum level and genera levels between control and SARA cows. Zero and 8 weeks after feeding with HCD, the rumen fluid, milk, and feces samples were collected for analysis bacterial community at phylum levels and genera levels. (A) Relative abundance of the top 10 phyla of milk, rumen fluid, and feces bacteria between control and SARA cows. (B) Relative abundance of the top 10 genera of milk, rumen fluid, and feces bacteria between control and SARA cows.

The top 10 phyla and the top 30 genera in relative abundance were used to analyze the bacterial community structure of rumen fluid, milk, and feces of cows. At the phylum level, *Proteobacteria*, *Firmicutes*, *Bacteroidetes*, and *Tenericutes* were the four dominant phyla in all samples by relative abundance (Fig. 7A). *t* Test analysis showed that *Proteobacteria* was significantly increased in the rumen fluid microbiota, whereas *Firmicutes*, *Tenericutes* and other low abundance phyla, including *Spirochaetes*, *Gracilibacteria*, *Euryarchaeota*, *Fibrobacteres*, and *Kiritimatiellaeota* were significantly reduced in SARA cows compared to control cows (see Supplementary Figure S4A in the supplemental material). The changes in the milk microbiota were similar to the changes in the rumen microbiota, as shown by the significant increase in *Proteobacteria*, while other phyla, including *Firmicutes*, *Bacteroidetes*, *Tenericutes*, and *Actinobacteria*, were significantly reduced in SARA cows compared to the controls (see Supplementary Figure S4B). In feces, the relative abundance of *Proteobacteria* was significantly increased, while the relative abundance of *Melainabacteria* was significantly reduced in SARA cows compared to control cows (see Supplementary Figure S4C).

In addition, the sequence results showed that *Stenotrophomonas*, *Bacteroides*, *Sphingononas*, *unidentified Ruminococcaceae*, and *Succinivibrio* were the most prominent genera in all samples (Fig. 7B). Significant differences in the abundances of milk, rumen fluid, and feces genera between control and SARA cows were further assessed by *t* test analysis. The results showed that *Stenotrophomonas* and *Succinivibrio* were markedly increased, while the other 10 genera were significantly reduced in rumen from SARA cows compared to those from control cows (see Supplementary Figure S4D in the supplemental material). The changes in the milk microbiota were also similar to the changes in the rumen microbiota, as shown by the relatively increased abundances of *Stenotrophomonas*, *Sphingomonas* and *Brevnudimonas*, while the other detected 14 genera were notably reduced in milk from SARA cows compared with those from control cows (see Supplementary Figure S4E). The microbiota in feces indicated that *Stenotrophomonas*, *Succinivibrio*, *unidentified Prevatellaceae*, *unidentified Erysipelotrichaceae*, *Unidentified Clostridiales*, *Roseburia*, *Acetitomaculum*, *Oscillibacter*, *Anaeroplasma*, and *Marvinbryantia* were significantly increased, and the other five genera were reduced in the SARA group (see Supplementary Figure S4F). These results showed that the changes in the microbiota of milk were similar to those in the rumen fluid samples after SARA. This indicated that there may be a certain correlation between the milk microbiota and rumen microbiota of dairy cows.

### *Stenotrophomonas* from the rumen may induce mammary gland inflammation in SARA cows.

**FIG 8:**
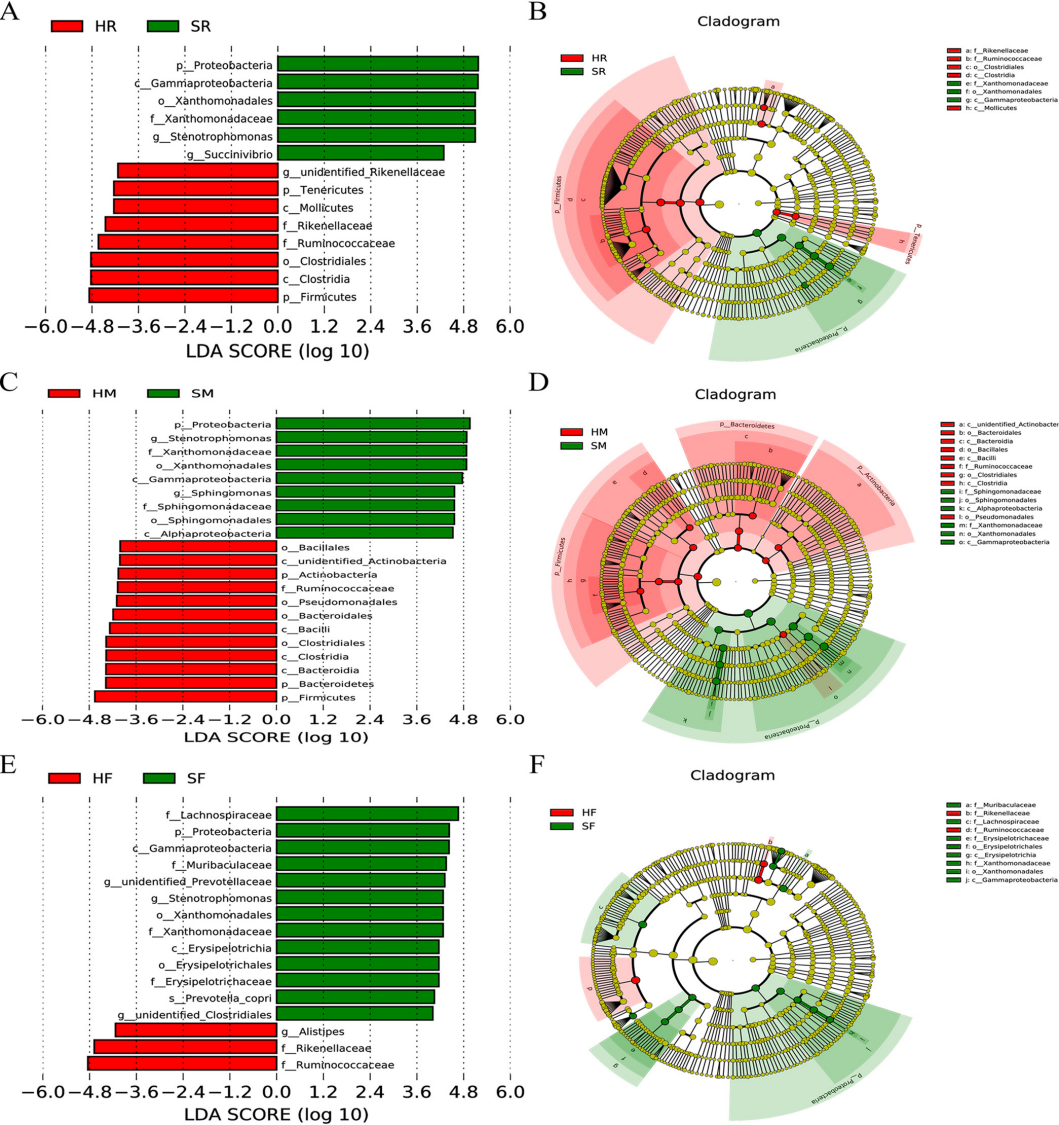
LEfSe analysis the microbiota from milk, rumen fluid, and feces between control and SARA cows. Linear discriminant analysis (LDA) score derived from LEfSe analysis, showing the biomarker taxa (LDA score of > 4 and a significance of *P *< 0.05 calculated by the Wilcoxon signed-rank test) in milk (A–B), rumen fluid (C–D), and feces (E–F) from control and SARA cows.

To identify the specific bacteria in the rumen associated with mammary gland inflammation, we performed a biomarker analysis using linear discriminant analysis (LDA = 4.0) effect size (LEfSe) and a cladogram generated from LEfSe analysis on the microbiota community of milk, rumen fluid, and feces. At the genus level, only *Stenotrophomonas* and *Sphingomonas* were enriched in milk from SARA cows compared to the control cows (Fig. 8A and B). Additionally, the relative abundance of *Stenotrophomonas* in rumen fluid and feces was significantly increased in SARA cows compared to control cows (Fig. 8C–F). These results suggested that a large amount of *Stenotrophomonas* derived from the rumen may be associated with the development of mastitis.

### Treatment of S. maltophilia-induced mastitis in mice.

**FIG 9:**
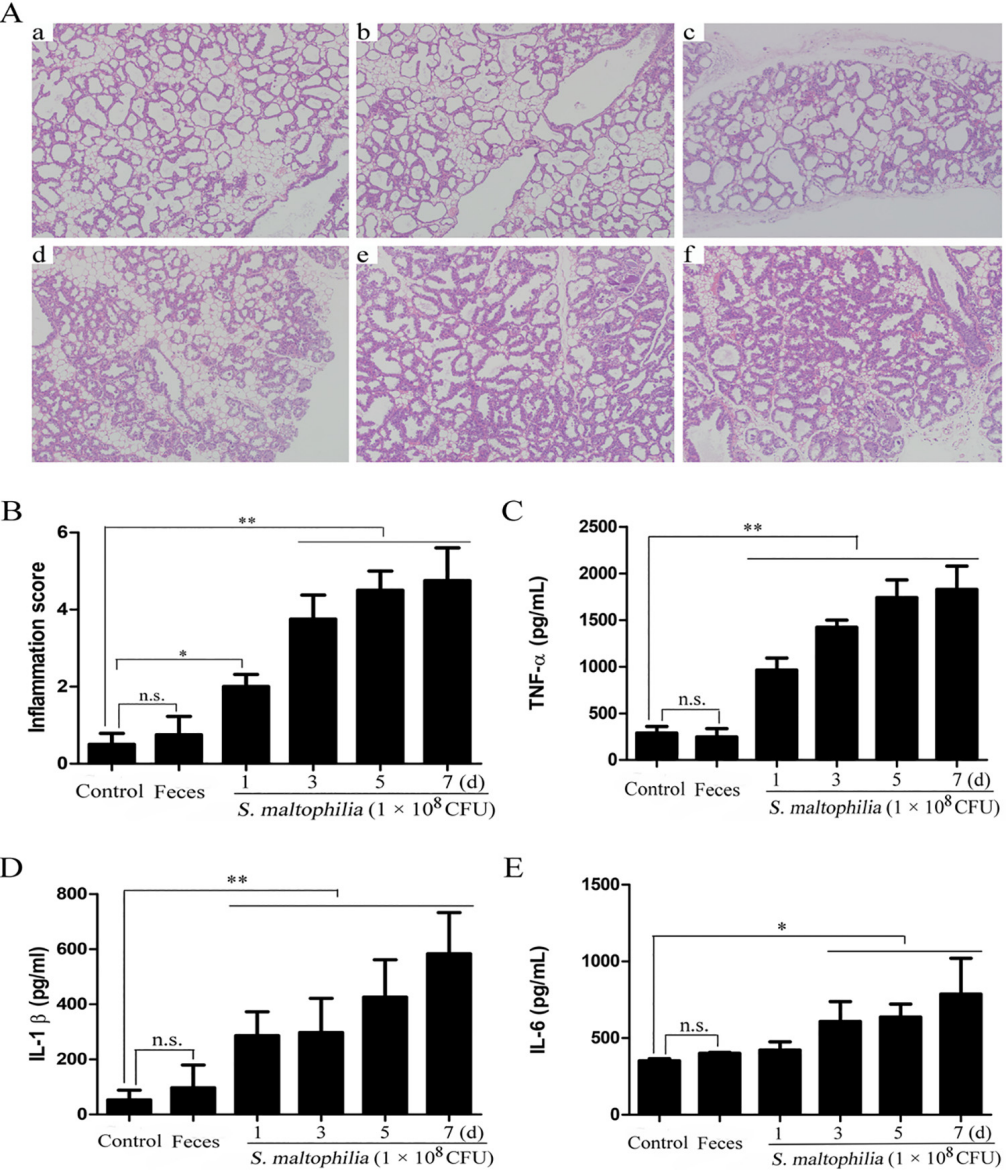
Treatment of *Stenotrophomona*s. maltophilia (S. maltophilia) induced mastitis in mice. S. maltophilia (1 × 10^8^ CFU/200 μL PBS) were administered to mice by oral gavage for 1 d, 3 d, 5 d, and 7 d consecutive days (once a day). In addition, fecal samples from each of the treatment of S. maltophilia was collected, mixed, suspended with sterile saline solution, and gently smeared on the mammary gland surface by swabs for consecutive 7 day. (A) Histological analysis and (B) inflammation score of the mammary gland among different treatment group mice. (C) TNF-α, (D) IL-1β, and (E) IL-6 levels in mammary gland among different treatment group mice. *P *< 0.05 indicates a significant difference between the different groups.

To explore the relationship of elevated *Stenotrophomonas* in the rumen of SARA cows and the incidence of mastitis, we detected the effect of S. maltophilia (the only species of *Stenotrophomonas*) on mastitis in mice. The results showed that oral infection of S. maltophilia resulted in damage to mammary gland tissues (Fig. 9A and B), and the production of the proinflammatory cytokines TNF-α, IL-1β, and IL-6 was significantly increased in the mammary glands (Fig. 9C–E). In addition, to detect whether S. maltophilia on the mammary gland surface can induce mastitis, the feces from mice that were gavaged with S. maltophilia were collected and smeared onto the surface of healthy mice. H&E staining and ELISA analysis showed that no inflammation was present in the mammary glands of the mice smeared with feces from the mice that were gavaged with S. maltophilia for the duration of the experiment (Fig. 9A–E). These results suggested that gut-derived S. maltophilia is an important agent to induce the development of mastitis.

## DISCUSSION

Mastitis affects nearly all lactating mammals and is generally thought to be an inflammatory response of the mammary gland caused by local infection by environmental pathogens. Over the last few years, several new important causative factors of mastitis have been revealed, such as gut microbiota (14). The typical characteristics of SARA cows include rumen microbiota disturbance, and a significant increase in mastitis has been reported in SARA cows. SARA is often caused by feeding a high-concentrate diet to maintain a high milk yield. Therefore, in most experiments, the SARA model is established by feeding with a concentrate to forage ratio of 7:3 in dairy cows (27, 28). Thus, in the present study, we assessed the effects and mechanisms of rumen microbiota on mastitis based on a SARA model induced by HCD feeding in cows using a 16S rRNA gene V4 region analysis on the IsonS^TM^XL platform. In addition, microbial changes may result from direct or indirect effects of lifestyle, dietary habits, genetics, or other factors that vary between individuals. To eliminate adverse effects due to interindividual variations, a self-comparative analysis was proposed to reduce selection biases and achieve more reliable results (29). Thus, in this study, the samples collected before treatment were used as a control group and compared with the following treatment procedures.

SCC is widely used as a parameter for diagnosing mastitis in cows. We found that SCC was significantly increased in milk from SARA cows compared to the control cows in this study. In addition, mammary gland tissues were damaged, and the levels of inflammatory cytokines, including TNF-α, IL-1β, and IL-6, in both milk and mammary gland tissues were significantly increased in SARA cows. In addition, the cows with higher SCC showed no clinical symptoms such as redness, swelling, heat or pain in the mammary gland. These results indicated that SARA induced the occurrence of subclinical mastitis in cows. Dairy cows are often fed HCD to meet the nutritional demands of high milk production. However, feeding HCD for a long period in cows often leads to the development of SARA, in which the rumen pH falls below 5.8 and then results in rumen microbiota dysbiosis (30). Once cows suffer SARA, a large amount of LPS is released into the rumen and translocated into the bloodstream across the rumen epithelium (31). As a result, the high level of LPS in the blood results in a systemic inflammatory response and metabolic alterations (32). During the lactation period, the mammary gland blood volume of cows accounts for 8% of the total body blood volume. It is suggested that, for every 1 L of milk secretion, the udder must have 400 to 500 L of blood flow, that is, 280 mL of blood flow every second. The large blood flow of lactating cows’ mammary glands suggests that LPS in the blood may circulate into the mammary gland and then induce inflammation of the mammary gland. Studies have also indicated that long-term overfeeding of an HCD accelerates rumen-derived LPS entering the blood circulation, inducing inflammatory and immune responses and increasing the synthesis of LAP by NF-κB signaling in the mammary gland of lactating cows (33). In addition, overproduction of proinflammatory cytokines activates the apoptosis pathway and induces mammary cell apoptosis in lactating cows (34). Increased amounts of LPS induced by SARA also affect milk fat and milk proteins by enhancing the methylation of genes relevant to fat synthesis and inhibiting the methylation of genes associated with protein synthesis (35, 36). We found that the levels of LPS in milk, mammary gland, lacteal vein, jugular vein, and rumen fluid were all markedly increased in SARA cows. The liver is an important immune and metabolic organ that has a strong ability to clear LPS (37). However, once increased LPS in the bloodstream enters the hepatic vein, portal vein, and artery, the clearance rate of LPS in the liver is significantly increased, but the percentage of removed LPS is reduced due to damaged hepatocytes elicited by the increased entry of LPS into the liver through the portal vein (24). Therefore, liver function was injured, as shown by increased levels of SAA, TNF receptor associated factor 6 (TRAF6), and MAPK (38), as well as inhibited oxidative status in SARA cows (39). We also found that SARA cows showed hepatocyte damage and inflammatory cell infiltration. AST, an important liver function marker, was also increased in SARA cows. These results suggest that LPS accumulates in the mammary gland through the bloodstream. In addition, the blood-milk barrier is a specific structure that plays an important role in preventing foreign matter, such as LPS, from the blood or external environment entering the mammary gland (22). Studies have shown that infusion of LPS disrupts the blood-milk barrier by regulating the expression of claudins in alveolar epithelial tight junctions through TLR4 activation (40). In the present study, cows that received SARA had reduced expression of tight junction proteins in the mammary gland. In addition, rumen and intestinal barrier permeability was also increased in cows suffering from SARA. The results suggested that SARA led to rumen-derived LPS entering the mammary gland through blood circulation, damaging the blood-milk barrier, and inducing inflammation of the mammary gland in cows.

The rumen microbiota is associated with mammary health in cows. Previous studies have demonstrated that the rumen bacterial community differs in cows with low and high SCCs (41). In addition, Ma et al. successfully induced inflammation of the mammary gland through transplantation of feces from mastitis cows to germfree mice, which indicated that intestinal microbiota is a vital cause of mastitis (14). Furthermore, our previous study showed that disturbance of the gut microbiota led to mastitis and increased the severity of mammary gland inflammation induced by S. aureus infection in mice (12). In this study, we found that rumen fluid, milk, and feces harbored distinct microbiota, and the bacterial community diversity and richness were both reduced in rumen fluid, milk, and feces during SARA induced by HCD in dairy cows. In addition, the community of bacterial flora in the rumen and milk of cows suffering from SARA were similar to those of control cows, both of which are characterized by a significantly increased abundance of *Stenotrophomonas* and associated with an increased risk of mastitis. In addition, mastitis was induced successfully by oral administration of *Stenotrophomona*s in the present study. *Stenotrophomona*s has been previously reported in cases of ruminant mastitis. Twenty buffalo milk samples isolated from subclinical mastitis caused by infection showed that S. maltophilia- induced mastitis accounted for 4% of cases (42). S. maltophilia was isolated from milk samples from an outbreak of bovine clinical mastitis (43). In addition, studies have shown that the level of *Stenotrophomonas* is increased in raw milk of SARA cows (44). These results suggested that there may be a close association between the rumen microbiota and milk microbiota, and the increased abundance of *Stenotrophomonas* in the rumen microbiota may be a risk factor for mastitis. These results suggested that the increased abundance of *Stenotrophomonas* in the rumen may be an endogenous trigger for mastitis.

An epidemiologic study of bovine *P. zofii* mastitis indicated that the disease may be associated with persistent infection in the gut and that the source of infection was feces. It has been suggested that microbial translocation occurs when *P. zofii* potentially disseminates from the gut to the mammary glands or other organs (45). A hematogenous route of bacterial infection cannot be discounted because SARA has been associated with liver abscesses (7), which are important causes of bacterial infection. Recent research has demonstrated that uterine pathogens can be transferred from the gut to the uterus via the bloodstream (46). In addition, studies have also indicated that circulating lymphoid cells containing engulfed bacteria are present in lactating mothers and labeled gut bacteria in lactating rodents are detected in breast milk (47). Furthermore, the mechanism by which gut bacteria enter the mammary gland clarifies that immune cells, especially dendritic cells (DCs), are involved in the translocation of gut bacteria into breast milk. DCs can retain live gut bacteria for several days and carry them to the mesenteric lymph nodes by passing through the lymphatic circulation. Then, these bacteria spread to distant organs, including the lactating mammary gland (48, 49). In the present study, the proportions of *Stenotrophomonas* in rumen fluid, feces, and milk were significantly increased in SARA cows. Whether dynamic changes in rumen microbiota and milk microbiota are associated with the translocation of pathogenic bacteria through blood should be assessed in the future.

In conclusion, our results demonstrated that rumen microbiota disturbance was associated with the occurrence of mastitis in cows. A possible mechanism for this may be that: LPS derived from the rumen can translocate to the mammary gland via the bloodstream and induce inflammation of the mammary gland. In addition, a high abundance of *Stenotrophomonas* in the rumen may lead to the development of mastitis. However, the molecular mechanism of mastitis induced by *Stenotrophomonas* in the rumen remains unclear and needs further study. Moreover, this study suggested that the tissues and organs of the body were interrelated, and diseases of local organs, such as the mammary gland, should be considered as a whole, especially maintaining the balance of the gut microbiota. This may be an important factor in the treatment of mastitis and other diseases. These results provide new theoretical guidance and an experimental basis for preventing mastitis by regulating rumen microbiota in dairy cows and also provide an important reference for the treatment of other infectious diseases in animal husbandry.

## MATERIALS AND METHODS

### Animals and experimental protocol.

Sixteen late-lactating Holstein Frisian cows (average body weight, 547 ± 51 kg, lactation days and similar weight) were used in this experiment. For 15 days before the start of the experiment, eight cows were offered free access to a diet containing a forage-to-concentrate ratio (F:C) of 40:60 to ensure adaptation to the diet. After 8 weeks, eight cows received a high-concentrate diet (HCD) comprising 30% forage and 70% mixed concentrate. Rumen fluid, milk, feces, and blood were harvested before feeding a HCD and were used as a control group for comparison following treatment with HCD cows. Throughout the experiment, the cows were fed daily at 5:00 and 18:00, and they had free access to water during the experimental period. The full proposal was reviewed by the Institutional Animal Care and Use Committee of the Jilin University ethics committee, which approved the animal care and use permit license. Experimental protocols for obtaining bovine clinical samples used in this study were carried out in strict accordance with the Animal Ethics Procedures and Guidelines of China. The sample collection work was approved with signed informed consent from the cattle farm owner.

### Sample collection and analysis.

Rumen fluid from each sample was collected by rumenocentesis using nonpyrogenic needles (2.0 × 120 mm) and 50 mL syringes at the beginning and after 8 weeks of feeding a HCD. The samples were centrifuged at 10,000 × *g* for 45 min at 4°C, and the supernatant was aspirated gently and passed through a disposable 0.22-μm LPS-free filter. The filtrates were transferred into sterile, depyrogenated glass tubes (Chinese Horseshoe Crab Reagent Manufactory Co., Ltd., Xiamen, China) and partly stored at −20°C for LPS detection. The others were frozen in liquid nitrogen and kept at −80°C for 16S rRNA rDNA gene amplicon pyrosequencing. Blood was collected from the jugular vein and lacteal vein at 0 and 8 weeks after feeding a HCD. Each sample was separated into two portions. One portion was collected with vacuum tubes containing heparin lithium and centrifuged at 3000 × *g* at 4°C for 15 min, and the plasma was harvested to detect the levels of ALT, AST, ALB, TP, and GLU using a biochemical analyzer (Vet Test 8008, IDEXX, USA). The second portion was collected with vacutainer tubes with EDTA. The plasma was separated from the blood by centrifugation at 3000 × *g* at 4°C for 15 min; transferred into a sterile, depyrogenated glass tube, and then kept at −20°C for LPS detection. Fecal samples were collected at the same time as blood and divided into two portions. One portion was immediately distilled with sterile PBS (1:5, w:v) to detect pH using a pH meter, and the second portion was collected and kept at −80°C for 16S rRNA gene amplicon pyrosequencing. The teats were disinfected using cotton wool dipped in 70% ethanol, and the first three handfuls of milk were discarded. Then, milk samples were collected from four quarters of each cow, mixed and divided into three portions from control and SARA cows. One portion was collected and transferred into sterile 50 mL vials with potassium dichromate. The samples were used to detect the milk composition (MilkoscanTM FT1, FOSS, Denmark) and SCC (Fossomatic 5000, FOSS). The second portion of the milk sample was collected and centrifuged at 14000 × *g* for 30 min at 4°C. The supernatants were transferred into a sterile, depyrogenated glass tube and kept at −20°C for LPS detection. The third portion was collected and kept at −80°C for microbiota analysis. Liver, rumen, and intestine tissues were collected from healthy and SARA animals after euthanasia. All samples were divided into two portions. One portion was fixed in 4% formaldehyde (Solarbio, Shanghai, China) for H&E staining (Solarbio, Shanghai, China), and another portion was kept at −80°C until use. The mammary gland tissues were collected and divided into three portions. The specimens were stained with H&E, kept at −80°C, and subjected to LPS detection. For LPS detection, 1 g of mammary gland tissues was weighed and homogenized with 0.5 mL of sterile PBS on ice, and the samples were centrifuged at 2000 r for 40 min at 4°C. The supernatant was collected and used for LPS detection.

### LPS analysis.

The levels of LPS from samples of rumen fluid, plasma, milk, and mammary glands were detected by a chromogenic endpoint assay (Chinese Horseshoe Crab Reagent Manufactory Co., Ltd., Xiamen, China) with a minimum detection limit of 0.1 EU/mL (rumen fluid and feces) or 0.01 EU/mL (plasma, milk, and mammary gland) according to the manufacturer’s instructions.

### Pro-inflammatory cytokines, LDH, IgG, and SAA assay.

Mammary gland tissues from control and SARA cows were weighed and homogenized with PBS (1:10, wt/vol) and centrifuged at 2000 × *g* for 40 min at 4°C. The lipids on the surface were removed, and the supernatants were collected. Milk was centrifuged at 1000 × *g* for 20 min at 4°C, and the supernatants were collected. Centrifugation was repeated, and the supernatant was kept at −20°C until use. The levels of TNF-α, IL-1β, IL-6, LDH, and IgG in plasma and milk were tested by ELISA kits (Lanpai BIO, Shanghai, China) according to the manufacturer’s instructions. SAA in the milk and plasma was detected using a biochemical analyzer (Diano, China).

### Western blot analysis.

The total proteins from the rumen, mammary gland, and intestine tissues were extracted with tissue protein extracted reagent (Thermo Scientific, MA, USA), and the concentration of proteins was detected using a BCA protein assay kit (Thermo Scientific). The proteins were fractionated by 10% sodium dodecyl sulfate-polyacrylamide gel electrophoresis (SDS-PAGE) and transferred onto PVDF membranes (Bio Trace, Pall Co., USA). The membranes were blocked with 3% BSA at room temperature at shaking for 2 h, and then incubated with anti-IκBα, anti-phospho-NF-κB p65, anti-NF-κB p65, and anti-TLR4 at 4°C overnight. Following tris-buffered saline with tween-20 (TBS-T) washing, all membranes were incubated with secondary antibody for 1 h. After washing with TBS-T. The targeted proteins were detected by Supersignal West Pico Chemiluminescent Substrate (Thermo Scientific), and the bands were imaged by a Protein Simple imager (ProteinSimple, Santa Clara CA, USA).

### Histopathologic analysis.

The tissues of the mammary gland, liver, rumen, and intestine of cows were histopathologically analyzed. Pathological injury scoring of liver tissue was conducted using a system that included three categories: glycogenated nuclei (graded 0–1, from none or rare to many continuous patches), liver cell ballooning injury (graded 0–2, from absent to severe ballooning injury), and inflammatory cell filtrate (graded 0–3, from none or rare to transmural) (38, 50). Rumen pathological injury was scored using a scoring system that included three categories: severity of epithelial injury (graded 0–3, from absent to mild) and the extent of inflammatory cell infiltrate (graded 0–3, from none or rare to transmural) (51). Each mammary gland index was evaluated through three categories, including hyperemia/edema (graded on 0 to 3, from normal to severe), milk stasis/acinar necrosis (graded on 0 to 3, from normal to severe), and infiltration with neutrophils (graded on 0 to 5, none or rate to transmural) (52). The intestinal injury score includes the severity of epithelial damage (scored 0–3, from none or rare to severe), the extent of inflammatory cell infiltrate (scored 0–3, from none or rare to transmural), and goblet cell depletion (sored 0–1) (53, 54). Three tissue sections from each sample were used to assess the numerical score.

### RNA extraction and quantitative real-time PCR (qRT-PCR) assay.

Total RNA of 0.1 g mammary gland, rumen, and intestine was extracted using TRLzol (Invitrogen, USA) according to the manufacturer’s instructions. RNA concentrations and quality were analyzed by an RNA/DNA calculator (Cambridge, UK). The total RNA of each sample was reverse transcribed into cDNA using random primers from a TransScript One-Step gDNA Removal kit and cDNA Synthesis SuperMix (TransGen Biotech, China) according to the manufacturer’s instructions. Quantification of relative mRNA concentration was conducted using a SYBR green Plus reagent kit (Roche, Swiss) and a 7500 Fast real-time PCR system (Applied Biosystems, USA). The relative expression of each gene was normalized to GAPDH, and the primers for each gene was shown in online Table S2.

### DNA extraction, 16S rRNA gene amplification, Ion S5TM XL sequencing, and data analysis.

Total genome DNA from rumen fluid, milk, feces, and blood was extracted by a CTAB/SDS method. The DNA concentration and purity of each sample were detected by 1% agarose gels and the concentration of DNA was diluted to 1 ng/μL by sterile water. The 16S rRNA was amplified by specific primer (16S V4:515F-806R) with the barcode targeting the V4 region. All PCRs were carried out with Phusion High-Fidelity PCR Master Mix (New England Biolabs). PCR products were mixed with an equal volume of 1×loading buffer (containing SYB green) and subjected to electrophoresis on a 2% agarose gel for detection. PCR products were mixed in equidensity ratios, and the products were purified with Gene JET Gel Extraction Kit (Thermo Scientific). Sequencing libraries were generated using Iso Plus Fragment Library Kit 48 rxns (Thermo Scientific) following manufacturer’s instructions. The library quality was evaluated by a Qubit@ 2.0 Fluorometer (Thermo Scientific). Finally, the library was sequence on an Ion S5 XL platform and 400 bp/600 bp single-end reads were generated.

Quality filtering of the raw reads was performed under specific filtering conditions to obtain high-quality clean reads according to the Cutadapt (V1.9.1) quality control process. The reads were compared with the reference database using the UCHIME algorithm (UCHIME Algorithm) to detect chimeric sequences, and the chimeric sequences were removed to obtain clean reads. Sequence analysis was performed by Uparse software (Uparse v7.0.1001). Sequences with ≥97% similarity were assigned to the same OTUs. Measures of alpha diversity, including Chao1, ACE, Shannon, and Simpson, were used to evaluate the complexity of species richness and diversity for each sample. All of the indices were calculated with QIIME. In addition, beta diversity and weighted and unweighted UniFrac analyses were selected to evaluate differences in samples in species complexity and were calculated using QIIME software. Principal coordinate analysis (PCoA) of weighted UniFrac analysis was used to evaluate the distance among the rumen fluid, feces, and milk in control and SARA cows. LEfSe was conducted to identify bacterial taxa differentially represented between rumen fluid, feces, and milk microbiota from SARA and control cows. Venn diagrams were created to analyze the number of core genera in milk, rumen fluid, and feces in SARA and control cows using the principles of bioinformatics and evolutionary genomics.

### Mastitis induced by *Stenotrophomonas* in mice.

In the study of the effect of *Stenotrophomonas* on mastitis, Stenotrophomonas maltophilia (S. maltophilia), the only species in the genus of *Stenotrophomonas*, were administered to mice (1 × 10^8^ CFU/200 μL PBS) after delivery by oral gavage for 1 d, 3 d, 5 d, and 7 d consecutive days (once a day). In addition, 1 g fecal samples from each of the S. maltophilia was mixed together and then suspended in sterile saline solution. After thorough mixing and resting (to minimize the number of bacteria lost), 2 mL of the supernatant was collected and gently smeared on the mammary gland surface by swabs for 7 consecutive days (14). The control group mice were smeared with the same volume of normal saline. Each group contained 8 mice after delivery. Then, the mice were sacrificed, and mammary glands were collected and stored at −80°C until use.

### Statistical analysis.

Statistical analysis was conducted using GraphPad Prism 6.01 (GraphPad Software, Inc., San Diego, CA). All data are expressed as the means ± SEM. Differences between date means were determined using one-way ANOVA (Dunnett’s *t* test) and the two-tailed *t* test. A *P *< 0.05 was considered to be statistically significant.

### Data availability.

16S rRNA raw data from the study were deposited in the NCBI Sequence Read Archive (SRA) under accession no. PRJNA786003.
