# Supplementary material for: The Rumen Microbiota Contributes to the Development of Mastitis in Dairy Cows
Source: Microbiol Spectr. 2022 Feb 16;10(1):e02512-21. doi: 10.1128/spectrum.02512-21 (PMC8865570; doi:10.1128/spectrum.02512-21)
Supplement: SUPPLEMENTAL FILE 1 — Supplemental material. Download SPECTRUM02512-21_Supp_1_seq11.pdf, PDF file, 0.7 MB [file spectrum02512-21_supp_1_seq11.pdf]

**Supplementary materials**

**Table S1:** pH in the rumen fluid, feces, and blood.

Table S1 PH in rumen fluid, feces, and blood

| Parameter | Groups | | | | *P*-value |
|---|---|---|---|---|---|
| | Control | | SARA | | |
| | $\bar{x}$ | SEM | $\bar{x}$ | SE | |
| Rumen fluid pH | 7.59 | 0.21 | 5.71*** | 0.25 | <0.0001 |
| Time < pH 5.8, h/d | | | 6.7 | 0.19 | |
| Feces | | | | | |
| pH | 7.39 | 0.19 | 7.01* | 0.21 | 0.003 |
| Blood plasma pH | 7.74 | 0.04 | 7.73 | 0.09 | 0.860 |

**Table S2:** Sequence of primers used in current investigation in qRT-PCR

Table S2. Sequence of primers used in current investigation in qRT-PCR

| Gene | Primer | Sequence 5’>3’ | Product Size(bp) |
|---|---|---|---|
| Claudin-1 | Sense | AGCATGGTATGGCAATAGAATTG | 125 |
| | Anti-sense | CTCCCAGAAGGCAGAGAGAAG | |
| Claudin-3 | Sense | TGGGAGGGACTGTGGATGAA | 127 |
| | Anti-sense | GGATGGCGATGACGATGAG | |
| Occludin | Sense | CGCCATTTTCGCCTGTGT | 191 |
| | Anti-sense | GGAATCCCTTTGCCGCTCT | |
| ZO-1 | Sense | CCTGCTTGACCTCCCAAAAG | 184 |
| | Anti-sense | GTCCACGACACGGAACACCT | |
| GAPDH | Sense | ACCCCTTCATTGACCTTCACTAC | 184 |
| | Anti-sense | ACCACATACTCAGCACCAGCAT | |

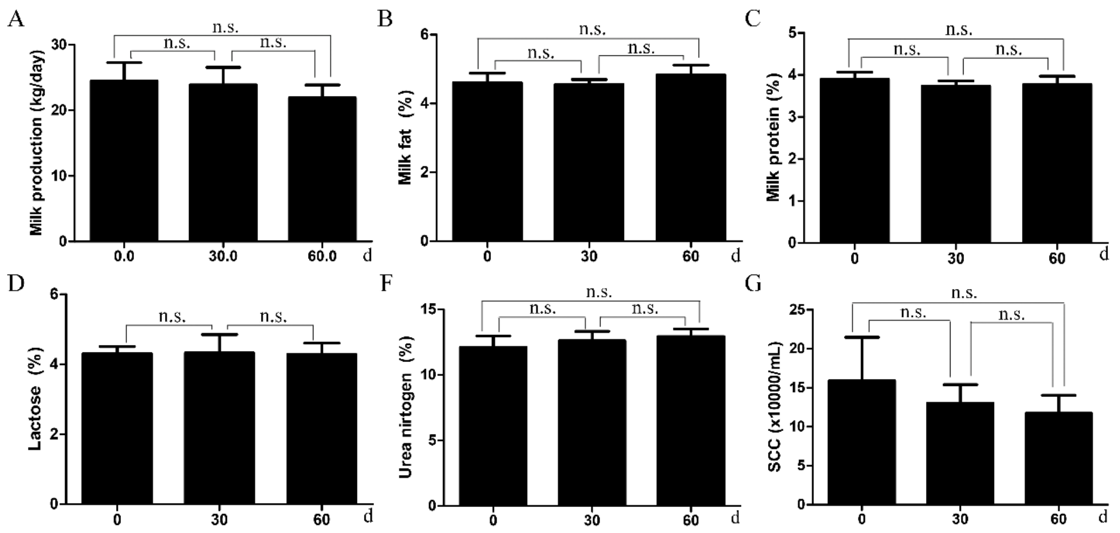


**Figure S1. Effect of experimental period on the changes of milk composition.** (A) Milk production, (B) milk fat, (C) milk protein, (D) fat/protein ratio, (E) lactose, (F) urea nitrogen, (G) SCC of milk from the healthy cows throughout the experimental period. n.s. indicates a no significant difference between the different groups.

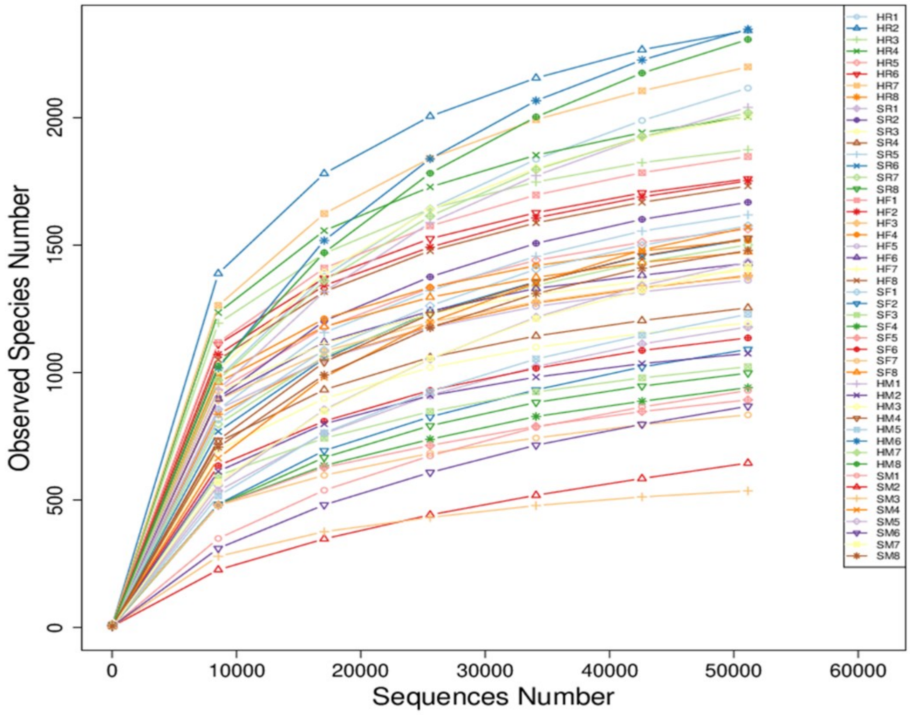


**Figure S2.** Rarefaction curves for 48 samples of rumen fluid, milk, and feces between control and SARA cows.

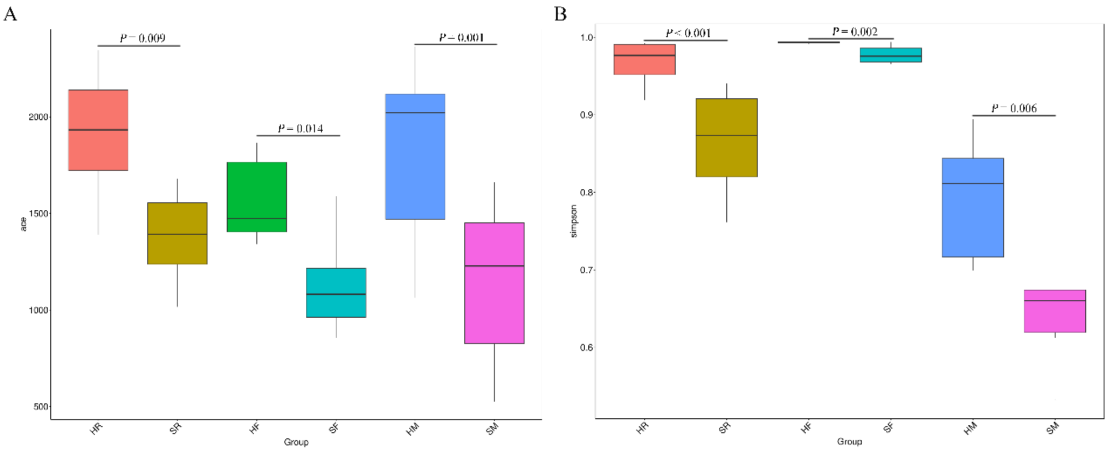


**Figure S3. SARA diminished the richness and diversity of the rumen fluid, milk, and feces microbiota.** Zero and eight weeks after feeding with HCD, the rumen fluid, milk, and feces samples were collected for analysis bacterial community. Comparison of the microbiota richness in terms of (A) ace in milk, rumen fluid, and feces between control and SARA cows. Comparison of the microbiota diversity in terms of the (B) simpson index in milk, rumen fluid, and feces between control and SARA cows. $P < 0.05$ indicates a significant difference between the different groups.

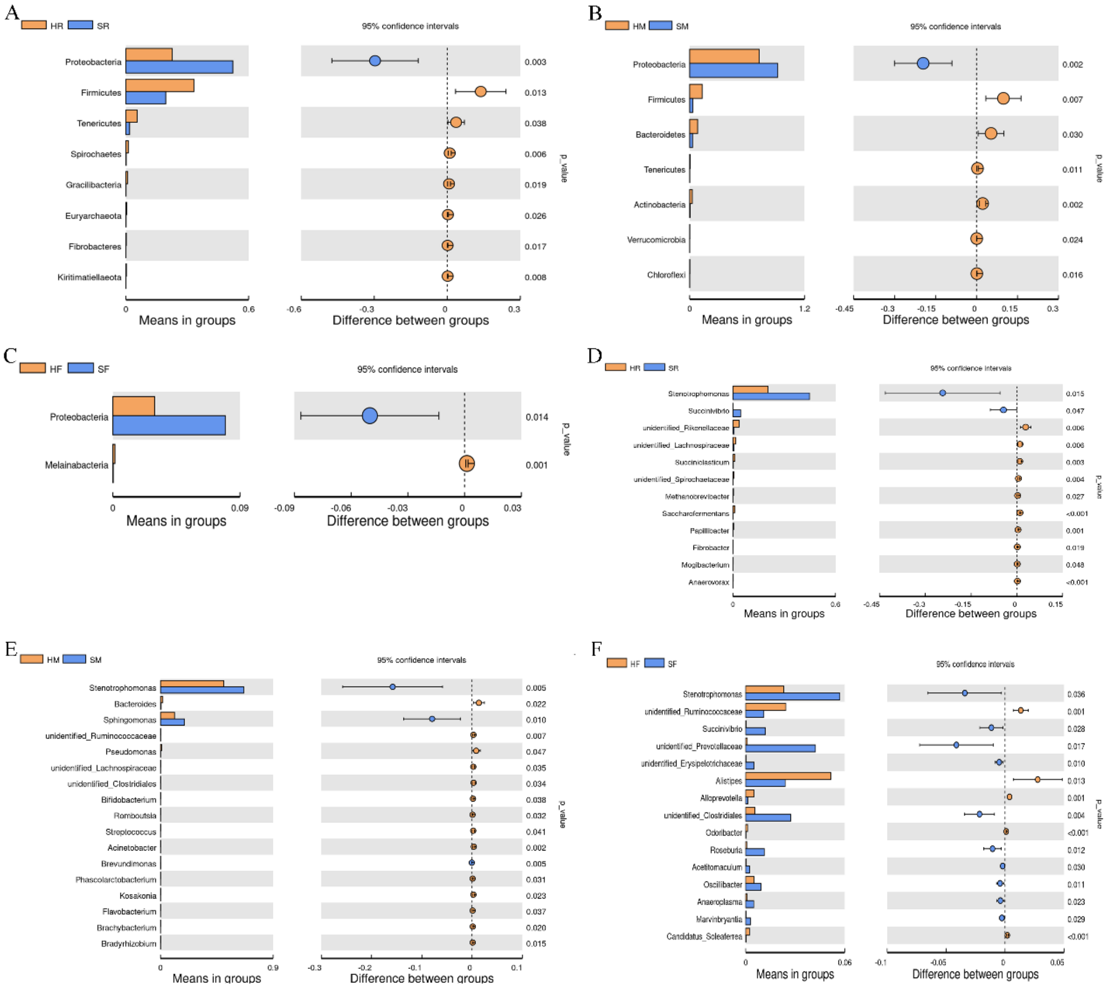


**Figure S4. Comparisons at the phylum level and genera levels between control and SARA cows.** Zero and eight weeks after feeding with HCD, the rumen fluid, milk, and feces samples were collected for analysis bacterial community at phylum levels and genera levels. T-test analysis of different bacteria in (A) rumen fluid, (B) milk, and (C) feces at the phylum level between the control and SARA groups. T-test analysis of different bacteria in (D) rumen fluid, (E) milk, and (F) feces at the phylum level between the control and SARA groups. $P < 0.05$ indicates a significant difference between the different groups.
